# Fore-Aft Ground Force Adaptations to Induced Forelimb Lameness in Walking and Trotting Dogs

**DOI:** 10.1371/journal.pone.0052202

**Published:** 2012-12-26

**Authors:** Jalal Abdelhadi, Patrick Wefstaedt, Ingo Nolte, Nadja Schilling

**Affiliations:** 1 University of Veterinary Medicine Hannover, Foundation, Small Animal Clinic, Hannover, Germany; 2 University of Tripoli, Faculty of Veterinary Medicine, Department of Surgery and Reproduction, Tripoli, Libya; 3 Friedrich-Schiller-University, Institute of Systematic Zoology and Evolutionary Biology, Jena, Germany; Friedrich-Schiller-University Jena, Germany

## Abstract

Animals alter their locomotor mechanics to adapt to a loss of limb function. To better understand their compensatory mechanisms, this study evaluated the changes in the fore-aft ground forces to forelimb lameness and tested the hypothesis that dogs unload the affected limb by producing a nose-up pitching moment via the exertion of a net-propulsive force when the lame limb is on the ground. Seven healthy Beagles walked and trotted at steady speed on an instrumented treadmill while horizontal force data were collected before and after a moderate lameness was induced. Peak, mean and summed braking and propulsive forces as well as the duration each force was exerted and the time to reach maximum force were evaluated for both the sound and the lame condition. Compared with the sound condition, a net-propulsive force was produced by the lame diagonal limbs due to a reduced braking force in the affected forelimb and an increased propulsive force in the contralateral hindlimb when the dogs walked and trotted. To regain pitch stability and ensure steady speed for a given locomotor cycle, the dogs produced a net-braking force when the sound diagonal limbs were on the ground by exerting greater braking forces in both limbs during walking and additionally reducing the propulsive force in the hindlimb during trotting. Consistent with the proposed mechanism, dogs maximize their double support phases when walking. Likely associated with the fore-aft force adaptations to lameness are changes in muscle recruitment that potentially result in short- and long-term effects on the limb and trunk muscles.

## Introduction

Animals have evolved behavioral plasticity to cope with a partial or total loss of a limb’s function. The associated lameness results from unloading the affected limb to relieve the pain, protect it from further damage and allow healing. Among other parameters such as kinematics or muscular recruitment, kinetic parameters change when a limb is unloaded and the animal adapts its locomotor mechanics to the new situation.

Ground reaction forces (GRF) are, for analytical reasons, described by orthogonal vectors; each component –mediolateral (Fx), craniocaudal (Fy), vertical (Fz)– having its own characteristic waveform and magnitude that reflect gait, speed and limb function. Therefore, many studies have analyzed GRF to determine limb function in clinically normal and abnormal dogs (e.g., forelimb function [1]–[9]). Of these studies, the overwhelming majority focused on the vertical force, because this component is easily measured using force plates or pressure pads and less susceptible to disturbances such as changes in speed or the direction of motion. Nevertheless, changes in the GRF vector will of course affect all three components [10]–[12]. Compared to the numerous studies on the changes in Fz in adaptation to lameness, very few studies have analyzed the craniocaudal component, which acts in the direction of body progression and results in fore-aft changes of the acceleration of the body. While several studies have described the fore-aft forces that dogs exert during normal gait (e.g., [12]–[26]), only very few studies have analyzed the changes in that GRF component due to lameness [3], [27], [28]. In those studies, adaptations in Fy in the affected and the contralateral forelimb in dogs with an unilateral fragmented coronoid process [3] or the two hindlimbs were reported because hindlimb lameness was the focus [27], [28]. Only one study has evaluated all limbs together and therefore was able to analyze the compensatory redistribution of fore-aft forces among the limbs; however, the dogs suffered from a total loss of a limb’s function due to amputation [29] rather than a partial impairment.

During steady-state locomotion, the forelimbs impart a net-decelerating force to the body and the hindlimbs exert a net-accelerating force, which have equal and opposite values [10], [12]. Because of this division of labor, the animal’s compensatory mechanism to a partial or total loss of a limb’s function can be expected to differ depending on whether a fore- or a hindlimb is affected. For example, in dogs with forelimb amputation, part of the braking impulse is assumed by the remaining forelimb [29]. Similarly, in dogs with hindlimb lameness, a greater share of the propulsive force is exerted by the sound hindlimb [27]. Furthermore, depending on the cause of lameness, the two phases of the fore-aft forces –braking (Fy-) and propulsion (Fy+)– may be affected differently. For example, it has been shown that in dogs with cranial cruciate ligament disease, although both phases were affected [27], the braking force was more affected than the propulsive force in order to avoid tibial thrust during early stance [30]. Dogs with a unilateral fragmented coronoid process showed more pronounced alterations in the propulsive than the braking force likely due to limited elbow extension during late stance phase [3]. Additional to changes in amplitude, the time spent exerting braking and propulsive forces may be altered. For example, when the function of a forelimb is lost, relative braking duration is significantly increased in the remaining forelimb [29].

During steady-state locomotion, the mean fore-aft acceleration for a complete stride is zero and no change in forward velocity occurs [10]. When animals accelerate or decelerate, a non-zero mean fore-aft force is exerted by the limbs that results in a moment about the pitch axis of the body [12]. That is, net-acceleration exerts a nose-up pitching moment about the center of body mass (CoM), while net-deceleration exerts a nose-down pitching moment. We hypothesized that these pitching moments due to changes in fore-aft acceleration may be one means by which animals temporarily unload an injured leg. Trotting animals with forelimb lameness may thus produce a nose-up pitching moment about the CoM by exerting a net-accelerating force when the lame diagonal limb pair is on the ground. To maintain steady state for a given stride, an opposing net-decelerating force resulting in a nose-down pitch should to be exerted when the sound diagonal limb pair was in ground contact. No previous study has evaluated the fore-aft force changes due to partial lameness for all four limbs, therefore it remains open whether lame animals produce a moment about their pitch axis (i.e., do a ‘wheelie’) to unload the affected forelimb or not.

The aims of this study were to 1) evaluate the changes in the fore-aft forces in all four limbs that are associated with a moderate forelimb lameness, 2) determine whether the braking or the propulsive forces are affected more by a distal load-bearing lameness, and 3) test the hypothesis that dogs produce a nose-up pitching moment about the CoM by exerting a net-accelerating force when the lame diagonal limb pair is on the ground. For heuristic reasons, we will focus on the craniocaudal component of the GRF in this study, although, as mentioned above, splitting the GRF into its orthogonal vectors is primarily a conceptual tool.

## Materials and Methods

### Animals

The present study is part of a larger investigation, which aimed at a better understanding of the compensatory mechanisms of walking and trotting Beagles with induced load-bearing forelimb lameness. Therefore, the same subjects and experimental design as in the previous study were used (for details, see [31]). Briefly, seven adult and clinically sound Beagles (3 males, 4 females) were used herein (age: mean ±SD: 6.6±3.0 years, 16.5±2.8 kg). All experiments were carried out in strict accordance with the German Animal Welfare Guidelines and were approved by the Ethics Committee of Lower Saxony, Germany (No. 12/0717).

### Experimental Design

The dogs walked and trotted on a horizontal treadmill at their preferred speeds, which ranged between 0.7–0.9 m/s and 1.2–1.4 m/s, respectively. Note that the trot was defined mechanically (i.e., according to [32]) despite the forelimbs showing a relative stance duration of duty factor >0.5 and thus this faster gait is a fast walk from a kinematic point of view [33]. Control data were collected before a reversible and moderate forelimb lameness was induced (reduction in peak Fz 35.5±9.0%) by taping a small sphere (9.5 or 16 mm in diameter) coated with cotton under the paw of the right forelimb (for details, see [31]). After forelimb lameness was induced, the measurements were repeated.

### Data Collection

**Figure 1:**
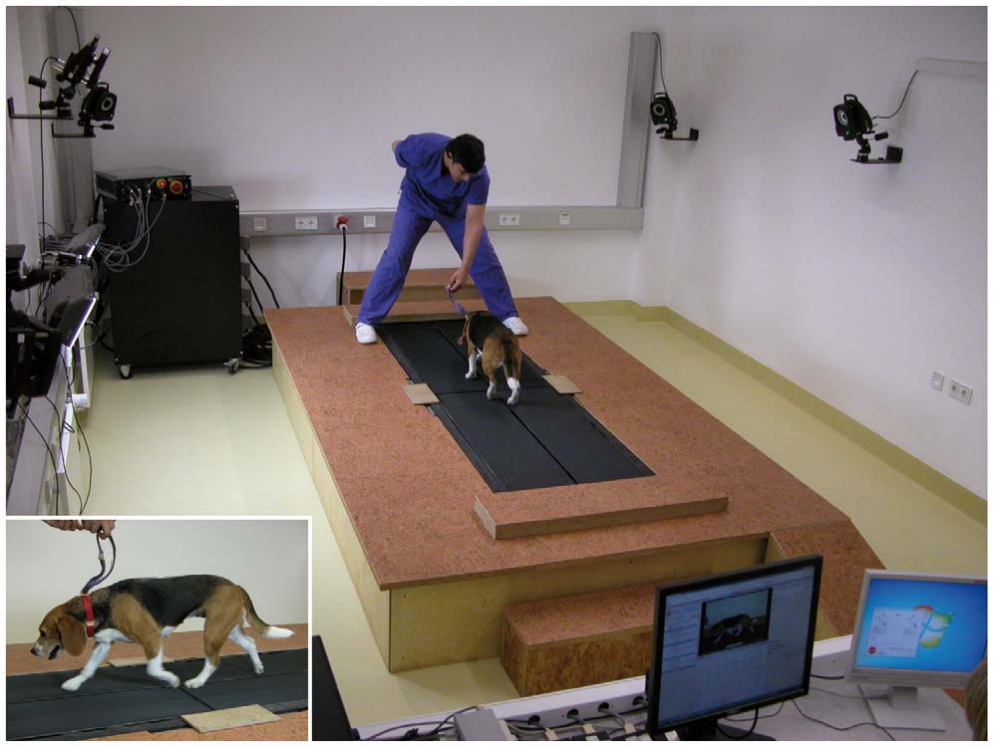
Experimental set-up used in the current study. The dogs walked and trotted at their preferred speed on the instrumented four-belt treadmill, which allowed for the synchronous collection of single limb forces before and after a distal load-bearing lameness was induced in the right forelimb (see inset).

Craniocaudal forces were measured using a four-belt treadmill system with a force plate implemented underneath each belt (Model 4060-08, Bertec Corporation; for technical details see [34]). Each belt has an approximate size of 30.5×122 cm, the subjacent force plates are 40×60 cm and are positioned as near as possible to the center of the treadmill system (Fig. 1). The instrumented treadmill is surrounded by a large wooden platform to provide enough space for the safe study of the animals. GRF data were collected at 1,000 Hz with a force threshold set at 13 N and processed using Vicon Nexus (Vicon motion systems Ltd.) and subsequently in Microsoft Excel 2003. At least five trials, each containing about 40 to 50 locomotor cycles, were recorded per dog. Data sampling started as soon as the dogs walked or trotted smoothly and comfortably (i.e., control data). After a rest of at least 15 min, the measurements were repeated with moderate lameness induced in the right forelimb. From both, control and induced-lameness data, the trials with the most regular gait and the greatest number of consecutive valid strides were selected per dog for further analysis. A stride was considered valid when each limb hit its respective force plate without overstepping onto the adjacent plate (i.e., when single limb forces were recorded; Fig. 1). This was evaluated using the digital video taken simultaneously from the lateral perspective (NVGS60, Panasonic) as well as the GRF data of all four plates.

### Data Analysis

The craniocaudal force was divided into its braking (Fy−) and propulsive (Fy+) component. In Vicon, touchdown and liftoff events of 10 consecutive strides were manually defined for each limb based on the vertical force curves. Because each stance phase varied slightly in duration and consequently in the number of recorded data points, the force curves were time-normalized to 100% stance duration (i.e., 101 bins) using linear interpolation. These time-normalized force data (in N) were averaged across the ten strides to obtain mean±SD for each dog and exported to Excel. Then, the data were normalized to the dog’s body weight using the following equation:
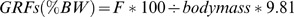
(1)


**Figure 2:**
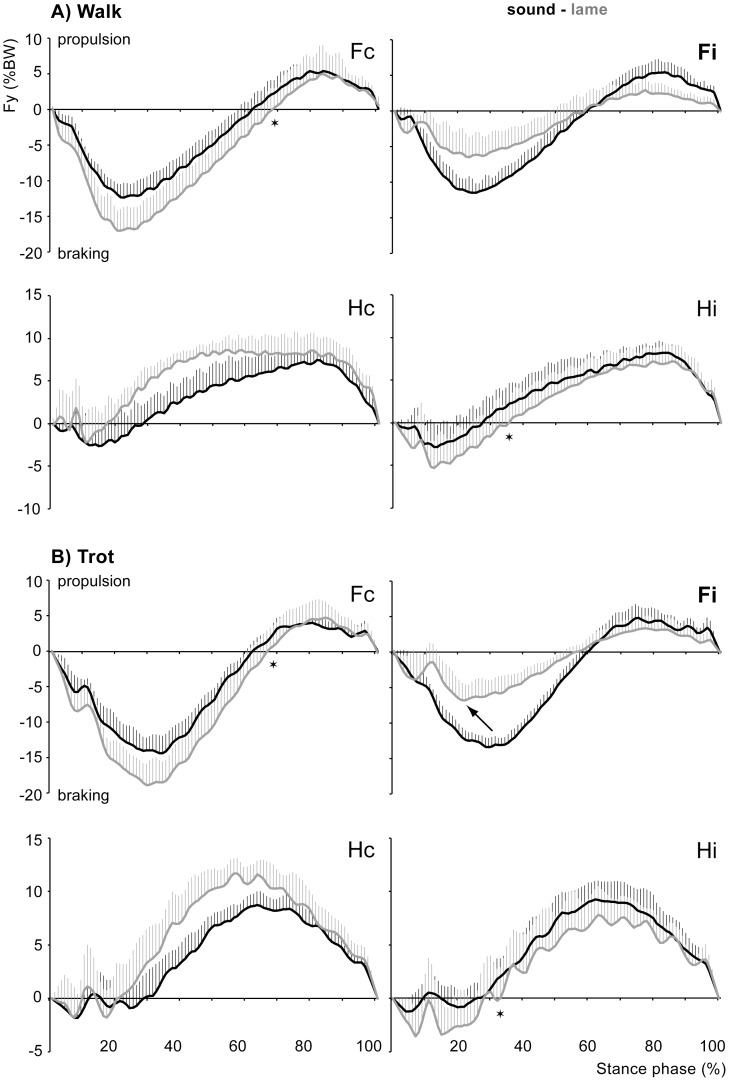
Fore-aft force profiles. Mean fore-aft forces (Fy in %BW) and SD (error bars) relative to stance duration in the sound dogs (black) and when lameness was induced (gray) in the ipsilateral forelimb (Fi) during walking (A) and trotting (B). The arrow indicates the only significant shift in the timing of PFy (p<0.05). The stars indicate significant shifts in the timing of the transition from braking to propulsive forces (p<0.05, see Tables 1 and 2 for the respective values). Fi = ipsilateral forelimb, Fc = contralateral forelimb, Hi = ipsilateral hindlimb, Hc = contralateral hindlimb.

Peak and mean force as well as the area under the curve (i.e., summed force) in percent body weight (%BW) were determined for Fy− and Fy+. We used summed force (in N) instead of impulse (in N/s) because the force data were time-normalized. Additionally, the duration during which braking force was applied and the time to peak force for braking and propulsion relative to the duration of the stance phase were determined. Note that because the braking force that the hindlimbs exert is small and may oscillate around zero before the propulsive phase begins, we determined braking duration by determining the last moment before the propulsion began (i.e., 100% stance phase minus duration of the propulsive phase; Fig. 2). Symmetry indices (SI) for both, the fore- and the hindlimbs, respectively were calculated (based on [35]):
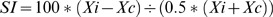
(2)


**Table 1:** Mean (M) and peak (P) as well as the sum (S) of the braking (Fy−) and propulsive (Fy+) forces in %BW for the sound condition and when lameness was induced in the right forelimb (Fi) in the walking Beagles.

	sound	lame		sound	lame	
	Fc			Fi		
PFy−	12.6±2.0	17.4±2.8	*	11.4±2.3	6.0±2.7	*
MFy−	6.7±1.2	8.5±3.7	*	6.4±1.0	3.7±1.6	*
SFy−	431.1±84.6	655.2±182.1	*	388.0±81.6	228.0±123.9	*
TPFy−	22.5±1.2	23.2±2.5	n.s.	23.6±2.2	24.2±3.5	n.s.
TFy−	62.1±4.1	68.3±5.7	*	59.9±4.0	58.3±10.5	n.s.
PFy+	5.8±1.7	5.7±3.6	n.s.	5.9±1.3	3.2±1.5	*
MFy+	3.5±1.0	3.0±1.9	n.s.	3.5±1.0	1.7±1.0	*
SFy+	139.6±56.1	109.8±83.5	n.s.	146.3±50.1	80.5±59.2	n.s.
TPFy+	83.5±2.8	88.3±6.5	n.s.	81.3±2.0	79.8±4.4	n.s.
	**Hc**			**Hi**		
PFy−	3.5±2.6	3.9±2.3	n.s.	3.9±2.1	5.8±2.8	*
MFy−	1.6±1.0	1.8±1.9	n.s.	1.8±1.0	2.8±1.7	*
SFy−	52.0±50.4	20.3±23.8	n.s.	49.5±37.0	104.9±83.1	*
TPFy−	9.9±5.0	6.6±3.3	n.s.	10.7±5.8	10.9±4.6	n.s.
TFy−	24.0±12.4	15.4±7.0	n.s.	26.7±8.8	34.3±8.9	*
PFy+	7.8±2.2	10.0±1.5	*	8.7±1.0	7.8±2.0	n.s.
MFy+	4.5±1.5	6.6±1.0	*	5.0±0.8	4.7±1.5	n.s.
SFy+	353.3±154.0	563.7±107.5	*	392.0±118.3	321.2±117.1	n.s.
TPFy+	80.0±3.3	64.0±14.8	n.s.	76.2±8.2	76.7±10.8	n.s.

Time to peak force (TPF) and the duration of the braking force (TFy−) are given in percent of stance duration. Significant differences between sound and lame conditions at * p<0.05; n.s. = not significant. Fi = ipsilateral forelimb, Fc = contralateral forelimb, Hi = ipsilateral hindlimb, Hc = contralateral hindlimb.

**Table 2:** Mean (M) and peak (P) as well as the sum (S) of the braking (Fy−) and propulsive (Fy+) forces in %BW for the sound condition and when lameness was induced in the right forelimb (Fi) in the trotting Beagles.

	sound	lame		sound	lame	
	Fc			Fi		
PFy−	14.7±2.2	19.4±2.9	*	13.7±1.0	7.4±2.6	*
MFy−	8.4±1.2	10.8±1.7	*	7.8±0.9	3.6±1.5	*
SFy−	505.9±100.5	738.4±172.3	*	465.5±61.9	215.5±96.0	*
TPFy−	31.8±3.4	31.5±3.0	n.s.	31.0±1.8	18.7±8.1	*
TFy−	61.3±3.7	67.0±5.9	*	59.9±3.0	58.3±4.7	n.s.
PFy+	4.6±1.0	5.6±2.2	n.s.	5.4±1.6	3.9±1.5	n.s.
MFy+	2.7±0.6	3.0±1.3	n.s.	3.2±1.0	2.2±1.0	n.s.
SFy+	107.9±28.9	109.0±60.9	n.s.	140.7±42.6	98.3±49.6	n.s.
TPFy+	84.6±6.7	84.4±5.8	n.s.	85.0±8.1	82.7±7.9	n.s.
	**Hc**			**Hi**		
PFy−	2.5±1.7	4.2±1.4	n.s.	2.2±1.1	4.1±1.7	*
MFy−	1.2±0.8	1.3±1.3	n.s.	1.0±0.6	2.0±1.5	n.s.
SFy−	31.9±32.1	28.8±31.1	n.s.	20.6±18.7	69.2±65.7	*
TPFy−	9.6±4.4	13.7±7.0	n.s.	8.8±6.5	12.7±5.8	n.s.
TFy−	27.7±9.8	24.3±5.5	n.s.	25.3±9.2	32.3±9.9	*
PFy+	9.1±1.1	12.6±1.3	*	9.7±1.7	8.6±2.0	n.s.
MFy+	5.1±1.0	7.2±0.8	*	5.3±1.1	5.0±1.5	n.s.
SFy+	406.2±91.78	540.4±127.6	*	440.0±112.7	357.3±134.4	*
TPFy+	66.0±8.4	59.4±7.0	n.s.	69.3±7.5	69.1±7.0	n.s.

Time to peak force (TPF) and the duration of the braking force (TFy−) are given in percent of stance duration. Significant differences between sound and lame conditions at * p<0.05; n.s. = not significant. Fi = ipsilateral forelimb, Fc = contralateral forelimb, Hi = ipsilateral hindlimb, Hc = contralateral hindlimb.

Thereby, X represents the mean value of peak, mean or summed force of the ipsilateral (i) and the contralateral (c) limb from the 10 strides.

To test whether the analyzed strides were at steady speed, we integrated the fore-aft force to determine the net-change in forward velocity of the CoM for each dog. This net-change in forward velocity was 5.2% of the total impulse produced by the dogs in the sound walking trials, 3.2% in the lame walking trials, 3.3% in the sound trotting trials, and 2.7% in the lame trotting trials. Except for the sound walking trials, in which a slightly greater net-forward acceleration occurred, all values were within the 5% range that has been found to be acceptable as steady-state locomotion in previous studies (e.g., [36]).

### Statistical Analyses

A Wilcoxon signed-rank test for paired observations was performed to detect differences in peak, mean and summed force as well as the temporal parameters between the sound and induced lameness conditions. P values p<0.05 were considered significant. All statistical tests were performed using GraphPad Prism (GraphPad Software, Inc.).

## Results

### Control Data

No significant differences in peak, mean and sum of the braking and propulsive forces were observed between the two fore- and the two hindlimbs during walking and trotting (Tables 1 and 2). Additionally, neither the relative time during which a braking force was applied nor the time to reach the maximum braking and propulsive force in percent of the stance duration were significantly different between the two forelimbs and the two hindlimbs when the dogs walked or trotted.

### Induced Forelimb Lameness Data

With regard to the braking component, peak, mean and summed force were significantly decreased in the forelimb in which lameness was induced (ipsilateral forelimb, Fi) and significantly increased in the contralateral forelimb (Fc) during both walking and trotting compared with the control data (Tables 1 and 2; Fig. 2). All three parameters were unchanged in the contralateral hindlimb (Hc) when the dogs walked and trotted. In the hindlimb ipsilateral to the affected forelimb (Hi), peak and summed force increased at both gaits, but mean force increased only during walking. Regarding the propulsive component, peak and mean force were significantly reduced in the ipsilateral forelimb during walking, but were unchanged during trotting. All three parameters were not significantly different from the sound condition in the contralateral forelimb and the ipsilateral hindlimb when the dogs walked and trotted (except summed force in Hi during trotting). In the contralateral hindlimb, peak, mean and summed force were significantly increased during trotting and walking.

During both walking and trotting, the time to peak braking and propulsive force was comparable between the sound and the lame conditions. The only significant difference was observed in the affected forelimb in trotting dogs; thereby, maximum braking force occurred significantly earlier during stance (Table 2; Fig. 2). The relative time to apply braking force was significantly longer in the contralateral forelimb and the ipsilateral hindlimb during both walking and trotting, while the transition from braking to propulsion occurred at about the same times in the affected forelimb and the contralateral hindlimb in sound and lame dogs (Fig. 2).

After lameness was induced, symmetry indices changed significantly for the peak, mean and summed braking force in the forelimbs during both walking and trotting, while they were unchanged for the propulsive force (except peak force during trotting; Table 3). In the hindlimbs, no significant changes were observed for the braking component. Regarding propulsion, the symmetry indices were significantly different for peak and summed force during walking, and peak and mean force during trotting after lameness was induced.

**Table 3:** Symmetry indices for mean (M) and peak (P) as well as the sum (S) of the braking (Fy-) and propulsive (Fy+) forces for the sound condition and when lameness was induced.

	Forelimb			Hindlimb		
Walk	sound	lame		sound	lame	
PFy−	−10.4±7.5	−100.6±31.1	*	22.7±52.4	27.7±60.5	n.s.
MFy−	−3.6±14.4	−69.6±46.0	*	22.8±54.1	58.4±136.8	n.s.
SFy−	−10.6±20.8	−99.6±49.6	*	18.5±70.3	108.7±106.2	n.s.
PFy+	3.4±17.9	−40.9±62.4	n.s.	13.7±33.0	−26.0±26.18	*
MFy+	0.2±15.4	−44.7±74.2	n.s.	13.6±47.9	−36.2±29.4	n.s.
SFy+	5.5±19.1	−18.3±94.4	n.s.	15.5±51.4	−58.4±24.2	*
**Trot**						
PFy−	−6.0±10.3	−89.9±36.0	*	3.4±102.7	−22.9±100.6	n.s.
MFy−	−4.9±12.1	−101.5±37.1	*	8.5±114.9	31.7±166.2	n.s.
SFy−	−7.9±10.8	−110.1±37.5	*	15.5±124.7	54.3±170.3	n.s.
PFy+	15.6±18.0	−31.4±54.0	*	5.1±17.2	−39.0±26.9	*
MFy+	14.7±24.8	−32.8±56.9	n.s.	3.2±20.5	−39.3±31.6	*
SFy+	20.2±20.1	−7.6±65.8	n.s.	6.9±27.3	−43.4±30.5	n.s.

Prefect symmetry is represented by SI = 0. Negative values indicate the respective parameter was greater for the contra- than the ipsilateral limb; positive values indicate a greater value for the ipsi- than the contralateral side. Significant differences between sound and lame conditions at * p<0.05; n.s. = not significant.

## Discussion

### Sound Condition

At steady state, the forelimbs impart a net-braking force while the hindlimbs impart a net-propulsive force (e.g., walking dogs [14], [21]; trotting dogs [12], [17]–[21], [24]). In the walking Beagles of this study, the forelimbs contributed about 90% of the total braking force exerted during the locomotor cycle and the hindlimbs exerted about 70% of the total propulsive forces. Compared with walking, this division of labor was even more pronounced during trotting; that is, the forelimbs contributed ca. 95% of the total braking and the hindlimbs ca. 80% of the total propulsive impulses exerted during a stride.

The evaluation of the net fore-aft acceleration showed that the dogs enrolled in this study walked and trotted at steady speed. Furthermore, the exerted braking and propulsive forces of the two fore- and the two hindlimbs were similar before lameness was induced. This together with the previous evaluation of the vertical load distribution among the limbs [31] shows that our dogs were sound and the collected data can serve as control data for the lame condition.

### Fore-aft Force Adaptations to Lameness

Compared to the sound condition, both braking and propulsion were reduced in the affected forelimb but the braking force was more affected than propulsive force during walking. This is in contrast to results from dogs with unilateral fragmented coronoid process disease, in which primarily the propulsive force was altered [3]. In the contralateral forelimb, the braking force was significantly increased in this study due to a greater amplitude but also a relatively longer time spent exerting a braking force. This agrees well with previous results in forelimb amputated dogs [29]. However, in contrast to the compensatory mechanisms of forelimb amputated dogs, in which both peak force and impulse of the braking component were significantly decreased in the ipsilateral hindlimb, in the dogs of this study, both the duration and the amplitude of the braking force were increased. In forelimb amputated dogs, both peak braking and peak propulsive forces were significantly decreased in the contralateral hindlimb [29], while in the dogs of this study, the propulsive force was significantly increased but the braking force was not changed. However, evaluating the specific relationship between the cause and degree of lameness and the observed force alterations is currently hindered, because too few studies have investigated fore-aft force changes in dogs with a unilateral loss of forelimb function.

During steady speed locomotion, braking and propulsive forces must be equal and opposing to prevent the body from undergoing a net rotation about its pitch axis [10], [12]. When comparing net fore-aft forces of the limbs of the sound diagonal pair with those of the lame diagonal pair in the sound condition, neither during walking nor during trotting a net-acceleration or net-deceleration was observed. Thus, the fore-aft forces exerted by the fore- and the hindlimb of a given limb pair were equal and opposing, resulting in pitch stability of the body at both gaits. In contrast, after lameness was induced, the lame diagonal limb pair exerted a net-propulsive force, while the sound pair exerted a net-braking force during both walking and trotting. Taken together, the exerted fore-aft forces for the two limb pairs were equal and opposing, providing pitch stability for a given locomotor cycle, but within the cycle the dog’s CoM underwent one acceleration and one deceleration. This results in a nose-up pitching moment at the CoM when the lame diagonal limb pair is on the ground and thus an unloading of the lame forelimb. To maintain balance and assure steady speed, the subsequent net-braking force produced a nose-down pitching moment when the sound diagonal limbs were in ground contact. In other words, within each locomotor cycle, the dogs did a ‘wheelie’ when the lame forelimb was in stance to reduce its loading and a ‘stoppie’ to restore pitch stability for that locomotor cycle.

A net-propulsive force in a diagonal limb pair can result from a reduced braking force of the fore- or the hindlimb or both, an increased propulsive force of the fore- or the hindlimb or both or a combination of thereof. Conversely, a net-braking force may result from increased braking and/or decreased propulsive forces of the diagonal limbs. Therefore, the amplitude and/or the time to apply the force may change. The results of this study show that in dogs walking and trotting at their preferred speed and with lameness induced in a forelimb, net-acceleration in the lame diagonal pair results from reduced braking forces in the forelimb with no changes in timing and increased propulsive forces in the hindlimb due to an earlier transition from braking to propulsion but also greater forces exerted by the limb. Net-deceleration in the sound limb pair during both walking and trotting was associated with greater braking forces in the fore- and the hindlimb due to both changes in timing and amplitude. Additionally, during trotting, the propulsive force exerted by the hindlimb was reduced.

Assuming that the locomotor adaptations to induced distal, load-bearing forelimb lameness observed herein are comparable to clinical load-bearing forelimb lameness, some implications follow from our results. In dogs with chronic forelimb lameness, the cyclic changes in fore-aft speed of the CoM are likely associated with muscular and potentially also skeletal stress of the locomotor system. For example, the nose-up pitch when the lame diagonal limbs are on the ground results, additionally to a lower braking force of the affected forelimb, from a greater propulsive force of the contralateral hindlimb. This, together with the greater moment at the hip, which has been proposed previously based on the increased vertical force exerted by this limb [31], must be accompanied by greater hindlimb retractor muscle activity, for example of the m. gluteus superficialis, m. semimembranosus or the m. biceps femoris [37], [38]. Because one of the main functions of the lumbar epaxial muscles in walking and trotting dogs is to stabilize the pelvis against extrinsic hindlimb muscle action [39], [40], the unilaterally greater forces acting on the pelvis may potentially lead to asymmetrical stress on the back muscles. Conversely, the nose-down pitching moment exerted to provide pitch stability for a given locomotor cycle results to a large degree from greater braking forces exerted by the forelimb of the sound diagonal limb pair. Associated with that, limb protractors such as the m. pectoralis superficialis descendens or the m. omotransversarius [38], [41] can be expected to increase their activity. In summary, the oscillations in fore-aft speed of the CoM are likely associated with substantial changes in muscular recruitment of a number of locomotor muscles and these muscular adaptations to lameness may potentially lead to faster muscle fatigue in the short run and be associated with changes in muscle size in the long run.

The trot is considered a two-beat gait in which, within each stride, two functional steps can be distinguished because the two diagonal limbs are closely coupled. This tight coupling likely facilitates the use of the herein proposed strategy of lame dogs ‘doing wheelies and stoppies’ because it enables the separate exertion of net-propulsive and net-braking forces by the diagonal limb pairs. During walking, however, for a good part of the cycle, three legs are on the ground. Three-legged ground contacts interfere with the production of a nose-up pitching moment as only for a short time the affected forelimb and the contralateral hindlimb are solely on the ground. Previous evaluation of the temporal gait parameters under the same experimental condition as used herein has shown that dogs walking with induced forelimb lameness significantly prolong the stance duration of the contralateral hindlimb and shift it forward relative to stride cycle so that the hindlimb’s touch down occurs earlier relative to the stance phase of the ipsilateral forelimb [31]. Additionally, the stance phase of the ipsilateral hindlimb shifts and accordingly the hindlimb lifts off earlier relative to the stance phase of the affected forelimb. In result, the time during which the lame diagonal limb pair is on the ground is prolonged and associated with that the time during which a nose-up pitching moment can be applied to unload the affected forelimb. Consistent with the diagonal sequence providing maximal overlap of the diagonal limbs, no changes in the temporal gait parameters were observed in forelimb lame trotting dogs [31].

### Conclusions

Additionally to the well-established adaptations in limb loading via changes in the vertical ground reaction force, this study shows that walking and trotting dogs with induced forelimb lameness also profoundly alter the craniocaudal force exerted by their limbs. By producing net-acceleration when the diagonal lame limb pair is in ground contact, dogs produce a nose-up pitching moment and thereby temporarily unload the affected forelimb. To regain pitch stability and ensure steady speed during the respective locomotor cycle, the produced net-deceleration results in a nose-down pitch when the sound diagonal limb pair is on the ground. As no clinical study has evaluated the fore-aft force adaptations to forelimb lameness in dogs, it remains open whether diseased dogs also use the proposed mechanism. Assuming they do, the likely associated changes in the recruitment of limb and trunk muscles will potentially lead to short- and long-term orthopedic consequences for the locomotor apparatus. Furthermore, our results show that evaluating the fore-aft component may be helpful in understanding the interdependencies between cause and degree of lameness and ground force adaptations.
